# NOTCH1 signaling in oral squamous cell carcinoma via a TEL2/SERPINE1 axis

**DOI:** 10.18632/oncotarget.27306

**Published:** 2019-11-26

**Authors:** Vasiliki Salameti, Priyanka G. Bhosale, Ashley Ames-Draycott, Kalle Sipilä, Fiona M. Watt

**Affiliations:** ^1^Centre for Stem Cells and Regenerative Medicine, King's College London, Tower Wing, Guy's Hospital, London, UK; ^*^These authors contributed equally to this work

**Keywords:** oral squamous cell carcinoma (OSCC), *NOTCH1*, *SERPINE1*, *ETV7*/TEL2, cell adhesion

## Abstract

Inactivating mutations in the EGF-like ligand binding domain of *NOTCH1* are a prominent feature of the mutational landscape of oral squamous cell carcinoma (OSCC). In this study, we investigated *NOTCH1* mutations in keratinocyte lines derived from OSCC biopsies that had been subjected to whole exome sequencing. One line, SJG6, was found to have truncating mutations in both *NOTCH1* alleles, resulting in loss of NOTCH1 expression. Overexpression of the NOTCH1 intracellular domain (NICD) in SJG6 cells promoted cell adhesion and differentiation, while suppressing proliferation, migration and clonal growth, consistent with the previously reported tumour suppressive function of NOTCH1 in OSCC. Comparative gene expression profiling identified *SERPINE1* as being downregulated on NICD overexpression and predicted an interaction between *SERPINE1* and genes involved in cell proliferation and migration. Mechanistically, overexpression of NICD resulted in upregulation of *ETV7*/TEL2, which negatively regulates *SERPINE1* expression. Knockdown of *SERPINE1* phenocopied the effects of NICD overexpression in culture. Consistent with previous studies and our *in vitro* findings, there were inverse correlations between *ETV7* and *SERPINE1* expression and survival in OSCC primary tumours. Our results suggest that the tumour suppressive role of *NOTCH1* in OSCC is mediated, at least in part, by inhibition of *SERPINE1* via *ETV7*.

## Introduction

Notch1 is a heterodimeric and multifunctional transmembrane receptor that regulates key cellular processes, including cell fate determination, maintenance of stem cells, cell survival, proliferation and apoptosis [1, 2]. Notch1 has an extracellular domain (NECD), transmembrane domain and intracellular domain (NICD) [3, 4]. Ligand mediated activation of Notch1 induces proteolytic cleavage and release of NICD, which translocates to the nucleus and activates transcription of downstream target genes [5].

NOTCH1 is frequently altered in cancer, resulting in aberrant activation or loss of signaling in a tissue and context dependent manner [6]. A comprehensive analysis of multiple head and neck squamous cell carcinoma (HNSCC) datasets demonstrated that 10–15% of HNSCC harbour inactivating *NOTCH1* mutations [7–9]. The majority of *NOTCH1* mutations occur in the EGF-like ligand binding domain of the NECD, and prevent ligand binding and downstream signaling [3].

The detection of *NOTCH1* mutations in dysplastic regions, and reduced expression of NOTCH1 in pre-neoplastic and cancerous skin lesions [10], suggests its potential gate-keeper properties. Some studies have implicated Notch1 signaling in angiogenesis and therapy resistance in HNSCC [11], while *in vitro* studies have pointed to the role of NOTCH1 in promoting keratinocyte differentiation [12]. Thus, it is important to understand how NOTCH1 contributes to oral tumorigenesis since it regulates multiple cellular processes and is a potential therapeutic target.

We previously performed whole exome sequencing of a panel of HPV-negative keratinocyte lines derived from oral squamous cell carcinomas (OSCCs), and identified *NOTCH1* mutations in several of the lines [13]. In the present study we have overexpressed NICD in a patient derived OSCC line with truncating mutations in both *NOTCH1* alleles. We provide evidence that the effects of NICD are mediated by negative regulation of serpin peptidase inhibitor, clade E, member 1 (*SERPINE1*) expression via *ETV7*. *ETV7* is a member of the ETS (E26 transformation specific) family of transcription factors and encodes TEL2, which plays a key role in cell migration and metastasis [14]. Thus, we provide new insights into the mechanism by which *NOTCH1* inactivation contributes to OSCC.

## Results

### *NOTCH1* mutations in OSCC lines

**Figure 1:**
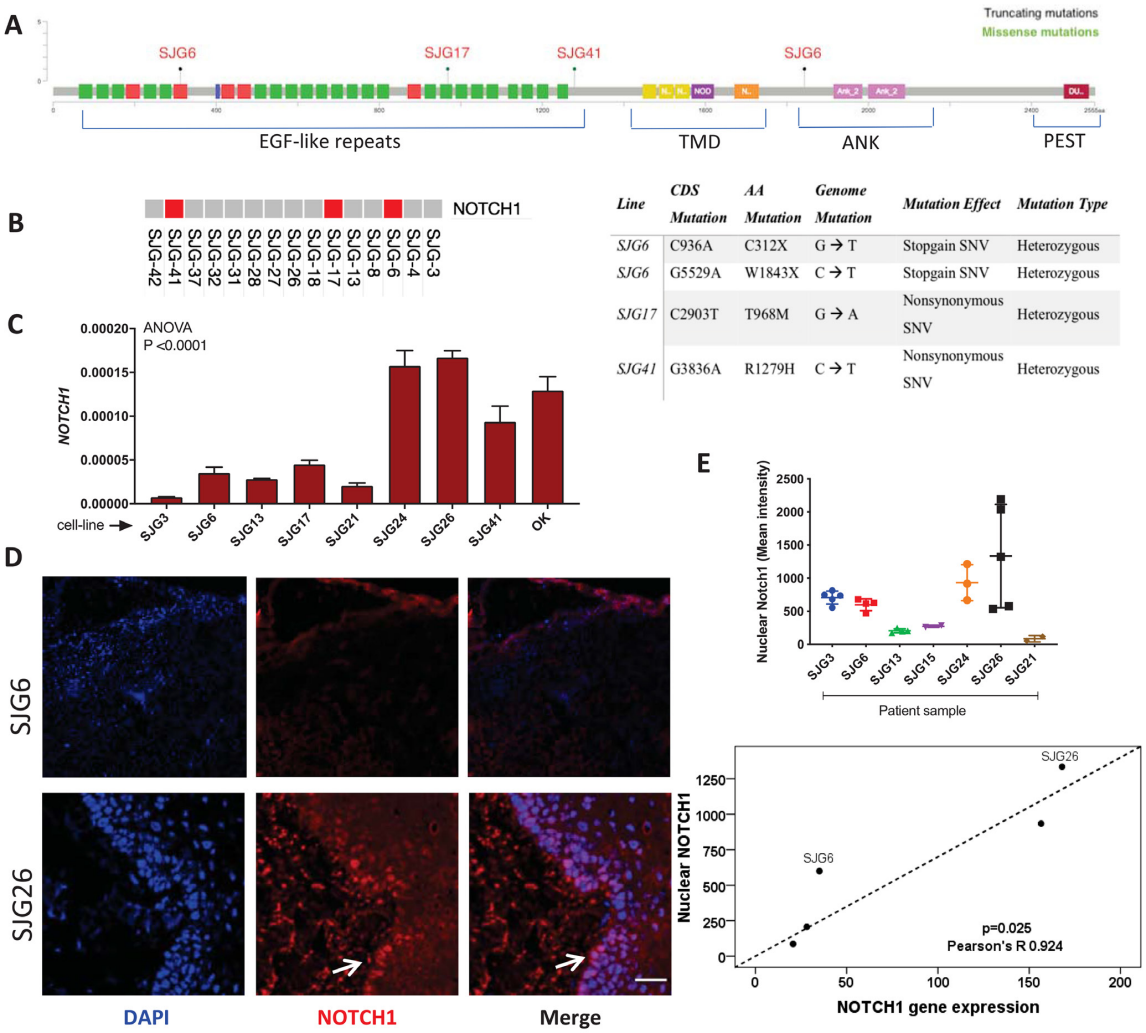
NOTCH1 expression in OSCC cell lines and parental tumours. (**A**) Schematic showing location of NOTCH1 mutations in SJG lines (red). EGF-like Repeats = epidermal growth factor-like repeats. TMD = transmembrane domain. ANK = ankyrin domain. PEST = proline, glutamic acid, serine, threonine-rich domain. Figure created using cBioPortal [61]. (**B**) Schematic showing which of the SJG cells lines have mutations (red), and the nature of the mutations (Table). (**C**) RT-qPCR analysis presenting levels of *NOTCH1* mRNA in SJG lines and oral keratinocytes (OK), *n* = 3. Data represent mean ± SD. (**D**) Immunostaining of SJG parental tumours for NOTCH1 (red, arrowed) with DAPI counterstain (blue). Scale bars: 100 μm. (**E**) Quantification of nuclear NOTCH1 mean staining intensity in SJG tumour biopsies (top). Data represent mean ± SD. Correlation between NOTCH1 nuclear staining intensity in parental tumours and *NOTCH1* mRNA expression in the corresponding SJG cell lines (bottom). *p* value was determined by Mann-Whitney test.

Based on whole exome analysis of 15 OSCC and the cell lines derived from them (Supplementary Table 1), we identified a hierarchy of nonsynonymous tumour specific mutations that was representative of mutations found in larger OSCC cohorts [13]. Three of the cell lines, SJG6, SJG17 and SJG41, harboured inactivating *NOTCH1* mutations, according to annotation in The Cancer Genome Atlas (Figure 1A, 1B) and were confirmed by Sanger sequencing (Supplementary Table 1). The expression of all 4 NOTCH receptors in the three lines that harbour NOTCH1 mutations was compared with normal oral mucosal keratinocytes (OK) and two OSCC lines that lack NOTCH1 mutations (Supplementary Figure 1A). There was no evidence that NOTCH1 mutations resulted in compensatory upregulation of *NOTCH2*, *3* or *4*; indeed SJG6 had the lowest levels of all 4 receptors.

To examine the effects of mutations on NOTCH1 expression, we performed real-time PCR of mRNA extracted from cell lines, and immunostaining for NOTCH1 in sections of the original tumours (Figure 1C, 1D). Compared to OK, there was reduced expression of NOTCH1 mRNA in the majority of OSCC lines, including SJG6 and SJG17 (Figure 1C). In those lines for which the original tumour was available (Figure 1D, 1E), there was a positive correlation between NOTCH1 mRNA expression and the mean intensity of nuclear Notch1 protein labelling in the corresponding tumour samples (R = 0.9241, *p* = 0.025) (Figure 1E, bottom panel). The difference in Notch1 expression between the tumours from which SJG6 and SJG26 were derived was particularly striking (Figure 1C–1E).

### Rescue of Notch signaling by NICD overexpression

**Figure 2:**
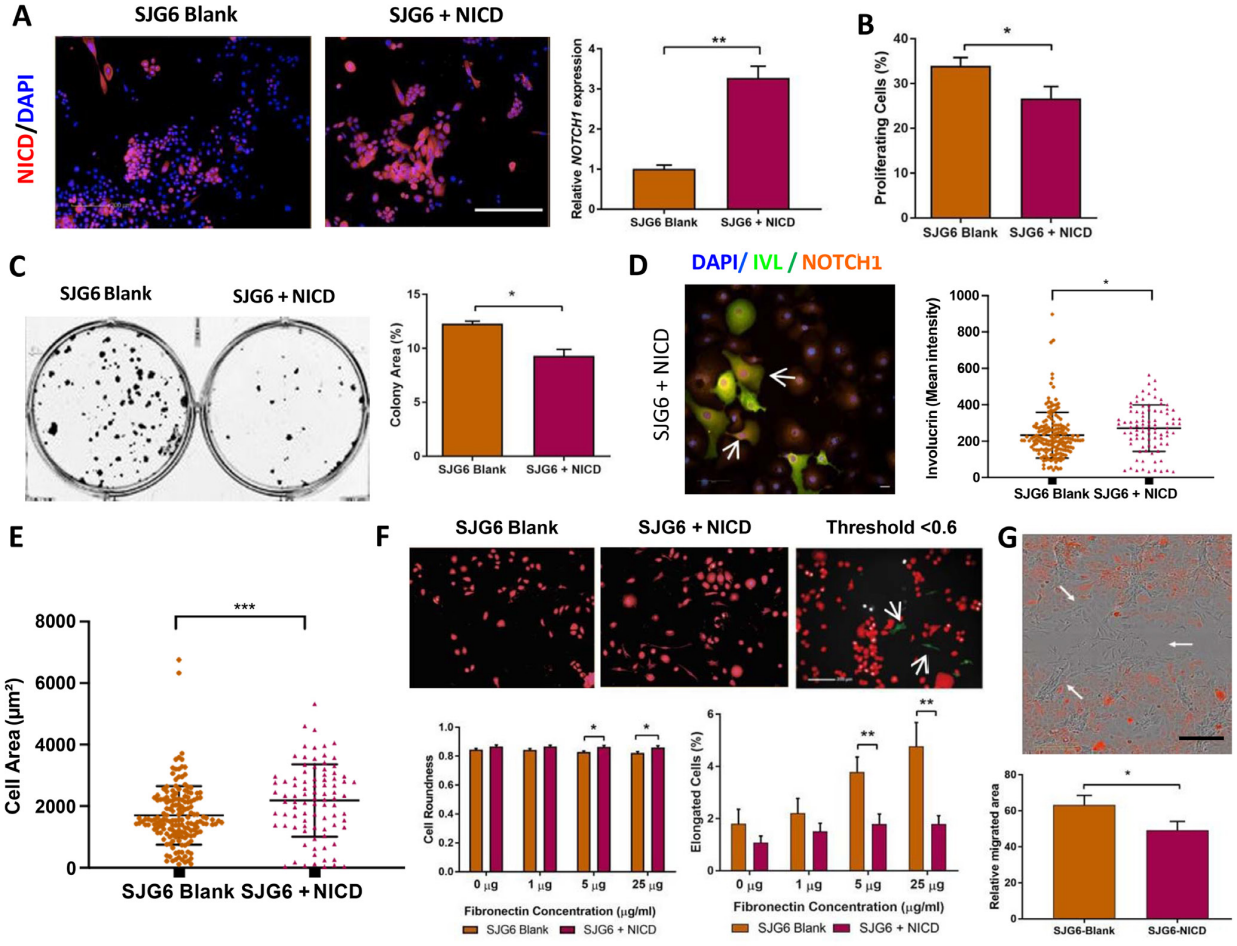
Phenotypic consequences of NICD overexpression. (**A**) mCherry (red) expression in SJG6 Blank and SJG6+NICD cells with DAPI counterstain (blue). Scale bar: 100 μm (left panels). RT-qPCR (right panel) showing relative expression of *NOTCH1* in SJG6 Blank and SJG6+NICD cells, *n* = 3. ^**^Student’s *t*-test *p* < 0.01. (**B**) Percentage of proliferating (EdU-positive) cells. *n* = 8. ^**^Student’s *t*-test *p* < 0.01. (**C**) Effect of NICD overexpression on clonal growth of SJG6 cells. Representative dishes and quantitation are shown. ^**^Student’s *t*-test *p* < 0.01. (**D**) Effect of NICD overexpression on differentiation. Representative image of Involucrin (green) and NOTCH1 (orange) immunostaining in SJG6+NICD cells and quantitation of mean staining intensity are shown. Scale bar: 20 μm. ^*^Multiple *t*-test, *p* < 0.05. (**E**) Increase in cell spreading on NICD overexpression. ^*^Multiple *t*-test, *p* < 0.05. (**F**) Quantitation of cell roundness and elongation via automated high content image analysis. In top panel images have overlay mask showing cells with a shape factor of less than 0.6 (red) and elongated cells (green). ^*^Multiple *t*-test *p* < 0.05; ^**^ *p* < 0.01. (**G**) Impact of NICD expression on cell migration. Representative image demonstrating migration of cells (arrowed) into the wound area (top panel) and quantitation of relative migration (bottom panel). Scale bar: 100 μm. ^*^Multiple *t*-test, *p* < 0.05. (A–G) Data represent mean ± SD.

Since the SJG6 line had inactivating mutations in both *NOTCH1* alleles (Figure 1A) and therefore low levels of NOTCH1 mRNA and protein, we chose this line to examine the effects of restoring Notch signaling. A lentiviral system was used to express NICD with an mCherry reporter (SJG6+NICD). A control line was generating by transducing SJG6 with mCherry alone (SJG6 Blank). NICD mRNA levels in SJG6+NICD were approximately 3-fold higher than in SJG6 Blank (Figure 2A) and thus similar to OK (Figure 1C). When SJG6 or OK expressing the highest levels of the NICD mCherry construct were flow sorted they did not proliferate on re-plating (data not shown). This is consistent with the role of NOTCH1 signaling in promoting keratinocyte terminal differentiation [13] and explains the modest level of NICD expression in those SJG6 cells that grew following transduction.

To measure the effects of NICD overexpression on cell behaviour, we performed assays of cell proliferation, differentiation, adhesion, migration and shape. We observed a significant reduction in cell proliferation, as measured by EdU incorporation (Figure 2B). This correlated with a reduction in colony formation (Figure 2C), consistent with the increase in keratinocyte proliferation that occurs on NOTCH1 knockdown [15]. NOTCH1 is a known regulator of keratinocyte differentiation [12], and, consistent with this, we detected a higher proportion of Involucrin-positive cells in SJG6+NICD than SJG6 Blank cultures (Figure 2D).

NICD expression also induced changes in cell adhesion, shape and migration [16]. SJG6+NICD cells exhibited a greater spread area 24 h after plating on collagen-coated flasks (Figure 2E). Furthermore we observed a reduced number of elongated cells and a greater number of cells with a rounded morphology in SJG6+NICD compared to SJG6 Blank cells plated on 5 or 25 μg/ml fibronectin (Figure 2F). When scratch wounds were made in confluent sheets of cells, there was faster migration of SJG6 Blank cells into the wound area than SJG6+NICD cells (Figure 2G). We conclude that loss of NOTCH1 enhances OSCC cell proliferation, colony formation and migration, while inhibiting differentiation and inducing changes in cell shape, consistent with previous reports [13, 15].

### NICD overexpression alters gene expression in SJG6 cells

**Figure 3:**
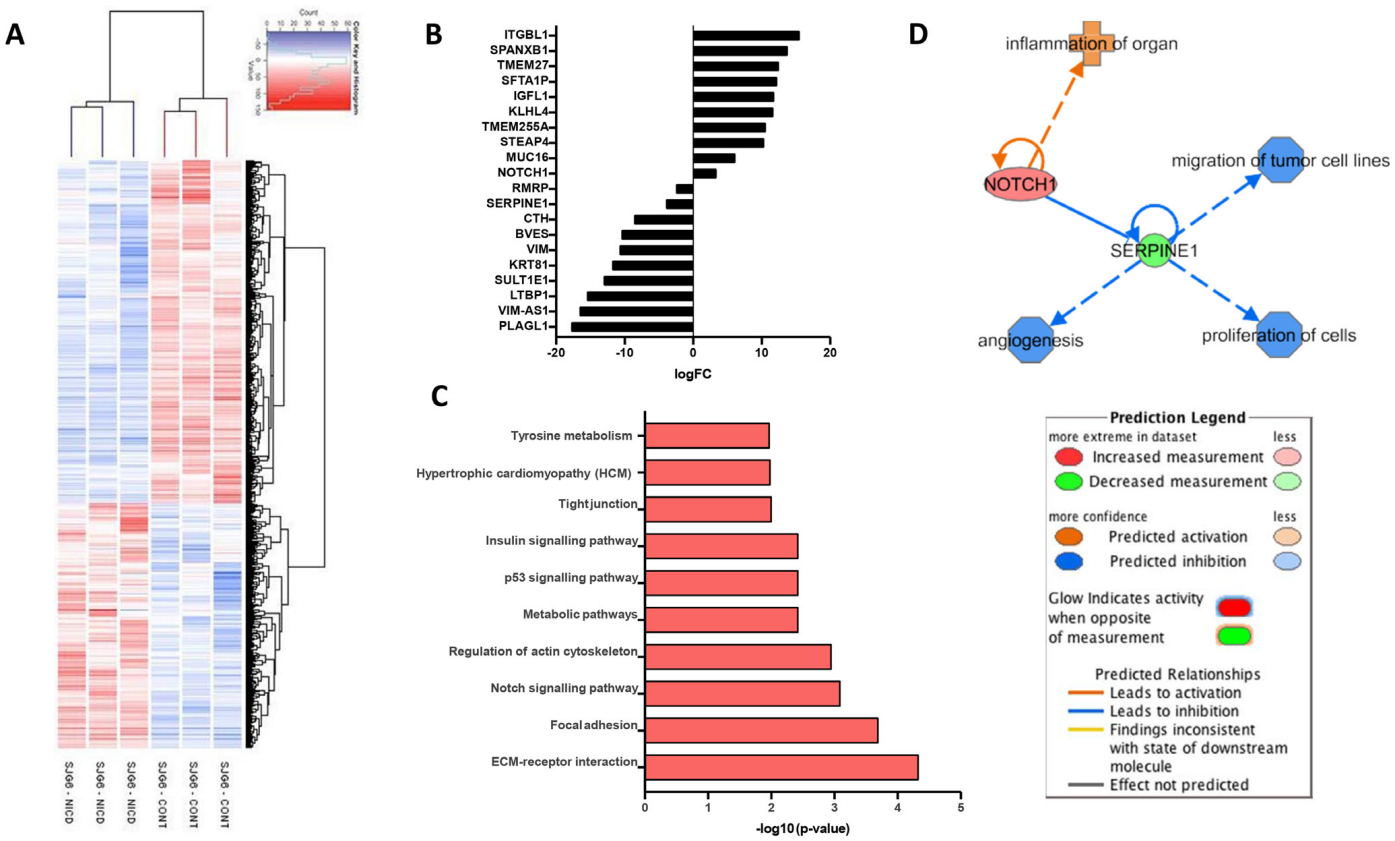
Gene expression profiling of SGJ6 cells transduced with NICD versus control. (**A**) Hierarchical clustering heatmap. (**B**) Top 20 up- or down- regulated genes in SJG+NICD cells. (**C**) Top 10 enriched KEGG pathways in SJG6+NICD cells. (**D**) Schematic created with IPA software showing SERPINE1 as a common regulator of NOTCH1 mediated cell behaviours.

To identify potential downstream targets of NOTCH1 we performed comparative gene expression profiling of SJG6 Blank and SJG6+NICD cells using an Affymetrix platform. We compared SJG6+NICD and SJG6 Blank cells from three independent lentiviral transduction experiments. SJG6 Blank cells clustered separately from SJG6+NICD cells (Figure 3A). The maximum fold change in *NOTCH1* expression was four-fold (Figure 3B, Supplementary Table 2). Enrichment of KEGG pathways in the gene set that was upregulated in SJG6+NICD cells included the NOTCH pathway itself, and genes involved in cell adhesion (Regulation of actin cytoskeleton, Focal adhesions, ECM-receptor interactions) (Figure 3C and Supplementary Table 3).

Of the top 20 genes found to be up- or down- regulated on NICD overexpression (Figure 3B), only *STEAP4* (Supplementary Table 3) is known to be a NOTCH target [17]. Of the top down-regulated genes, *PLAGL1, CTH* and *VIM* are positively correlated with HNSCC [18–20], while increased expression of *RMRP, SULT1* and *LTBP1* has been recorded in other cancers [21–23]. Therefore, our data are consistent with a role for NOTCH1 in altering expression of several OSCC genes (Supplementary Table 2). We also observed downregulation of the transcription factor *SNAI2*, which is known to promote an elongated cell shape, while expression of *TWIST1/2* and *ZEB1*, which promote EMT and metastasis, was reduced [24] (Supplementary Table 2). This is consistent with the changes in cell phenotypes that we observed on NICD expression in SJG6 cells.

We next examined the differentially expressed genes using Gene Set Enrichment Analysis (GSEA) [25, 26] for molecular and biological functions with an activation score of more than 2 (Supplementary Tables 4, 5). Of the 46 GO terms (Supplementary Table 4), 18 predicted that NICD expression is associated with a decrease in cell migration and 5 predicted a decrease in proliferation, consistent with the observed cellular behaviours (Figure 2). Microarray data were mined for genes that could link *NOTCH1* to the respective phenotypes, specifically reduced migration and proliferation and increased differentiation. *SERPINE1* was found to be common to all pathways (Figure 3D) and observed to be one of the top downregulated genes in SJG6+NICD cells (Figure 3B), suggesting a potential key role in mediating the effects of NOTCH1 signaling in OSCC. SERPINE1 (also known as PAI1) is a plasminogen activator inhibitor involved in the urokinase-type plasminogen activator system (uPA), and its binding to urokinase-type plasminogen activator receptor (uPAR) triggers signals that promote migration, proliferation and cell survival [27, 28].

### *SERPINE1* knockdown phenocopies NOTCH1 activation in OSCCs

Overexpression of *SERPINE1* in OSCC is correlated with increased cell migration, EMT and metastasis [29], while NICD expression has previously been linked to inhibition of *SERPINE1* in thyroid cancer [30]. It has been shown previously that downregulation of *SERPINE1* in nasopharyngeal carcinoma lines results in decreased cell migration and increased cell death [31]. To investigate whether *SERPINE1* mediates any of the phenotypes associated with *NOTCH1* mutation in SJG6 cells, we performed transient knockdown of *SERPINE1*. We predicted that when *SERPINE1* expression was silenced, the SJG6 cellular phenotypes would correspond to those observed upon NICD overexpression.

**Figure 4:**
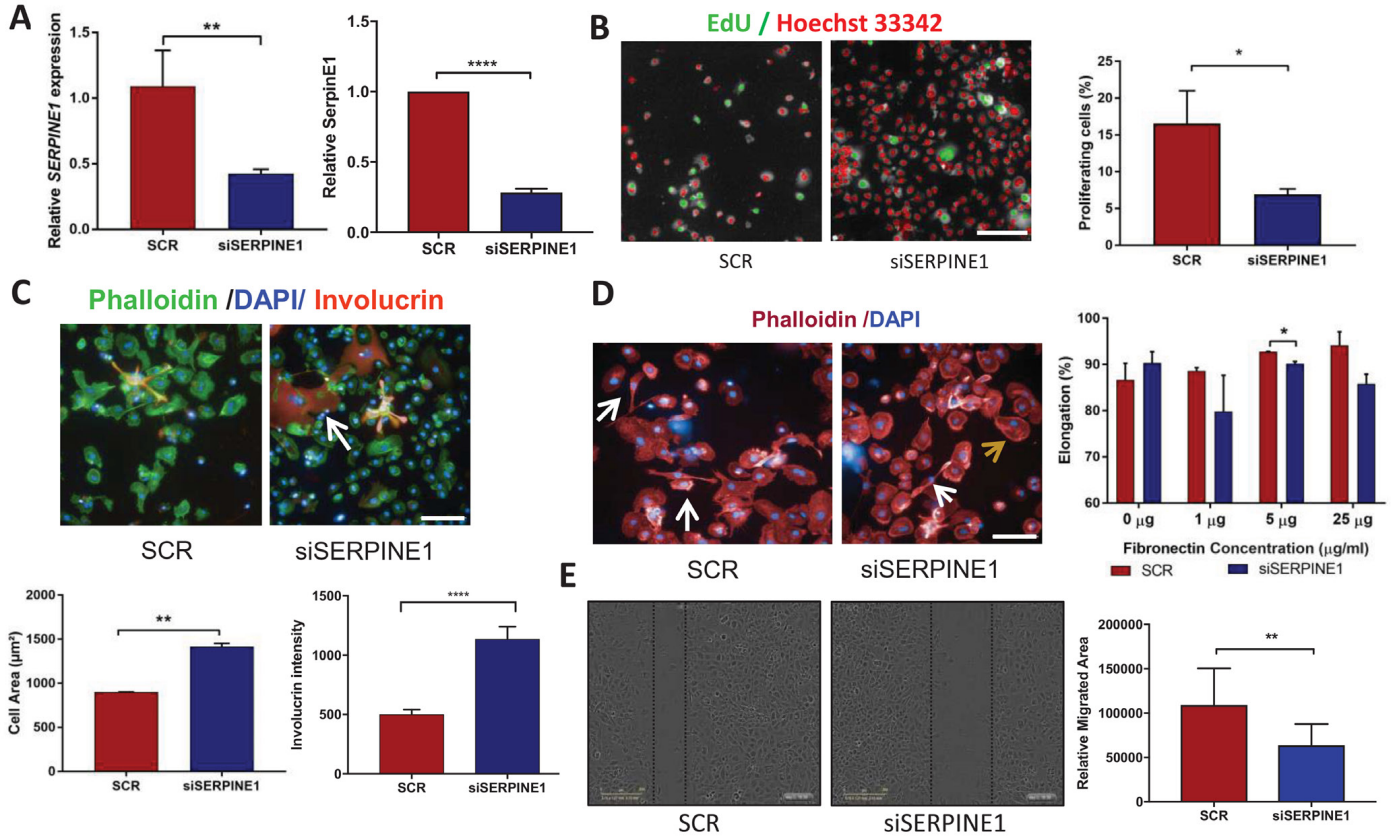
Cell phenotypes resulting from *SERPINE1* knockdown. (**A**) RT-qPCR (left) and Western blot densitometric analysis showing reduced SERPINE1 expression in siSERPINE1 cells compared to SCR controls. Multiple *t*-test, ^**^ *p* < 0.01; ^****^ *p* < 0.001. (**B**) Representative high content images after thresholding for Alexa 488 positive nuclei. EdU positive cells are depicted in green and negative cells in red, together with quantitation of % EdU labelled cells in SCR and siSERPINE1 (right). Multiple *t*-test, ^*^ *p* < 0.05. (**C**) Representative images showing Involucrin (orange; arrow) and Phallodin (green) staining with DAPI counterstain (blue). Changes in cell area and Involucrin labelling intensity per cell were quantified by high content imaging. Multiple *t*-test, ^**^ *p* < 0.01; ^****^ *p* < 0.001. (**D**) Representative images of elongated (white arrows) and non-elongated (brown arrow) cells plated on Fibronectin, together with quantification. Multiple *t*-test, ^*^ *p* < 0.05. (**E**) Scratch wound assays and quantitation of relative migration of SCR and siSERPINE1 cells. Multiple *t*-test, ^**^ *p* < 0.01. Scale bars: 100 μm. Data represent mean ± SD (A, E) or SEM (B–D).

siRNA mediated knockdown of *SERPINE1* was confirmed 72 h post-transfection in SJG6-siSERPINE1 (referred as siSERPINE1 cells) compared to SJG6-Scrambled (SCR) by RTq-PCR and Western blotting (Figure 4A). We observed a significantly higher percentage of proliferating cells in SCR compared to siSERPINE1 cells (Figure 4B). siSERPINE1 cells exhibited a significantly greater cell area and increased involucrin labelling intensity than SCR cells (Figure 4C) and the proportion of elongated cells was reduced on 5 and 25 μg/ml fibronectin (Figure 4D). Furthermore, in a scratch wound assay SCR cells had a higher migration rate than siSERPINE1 cells (Figure 4E). These results suggest that upregulation of *SERPINE1* on NOTCH1 inactivation could contribute to the changes in OSCC cell adhesion, migration, proliferation and differentiation.

### NICD represses *SERPINE1* expression by regulating *ETV7*/TEL2

**Figure 5:**
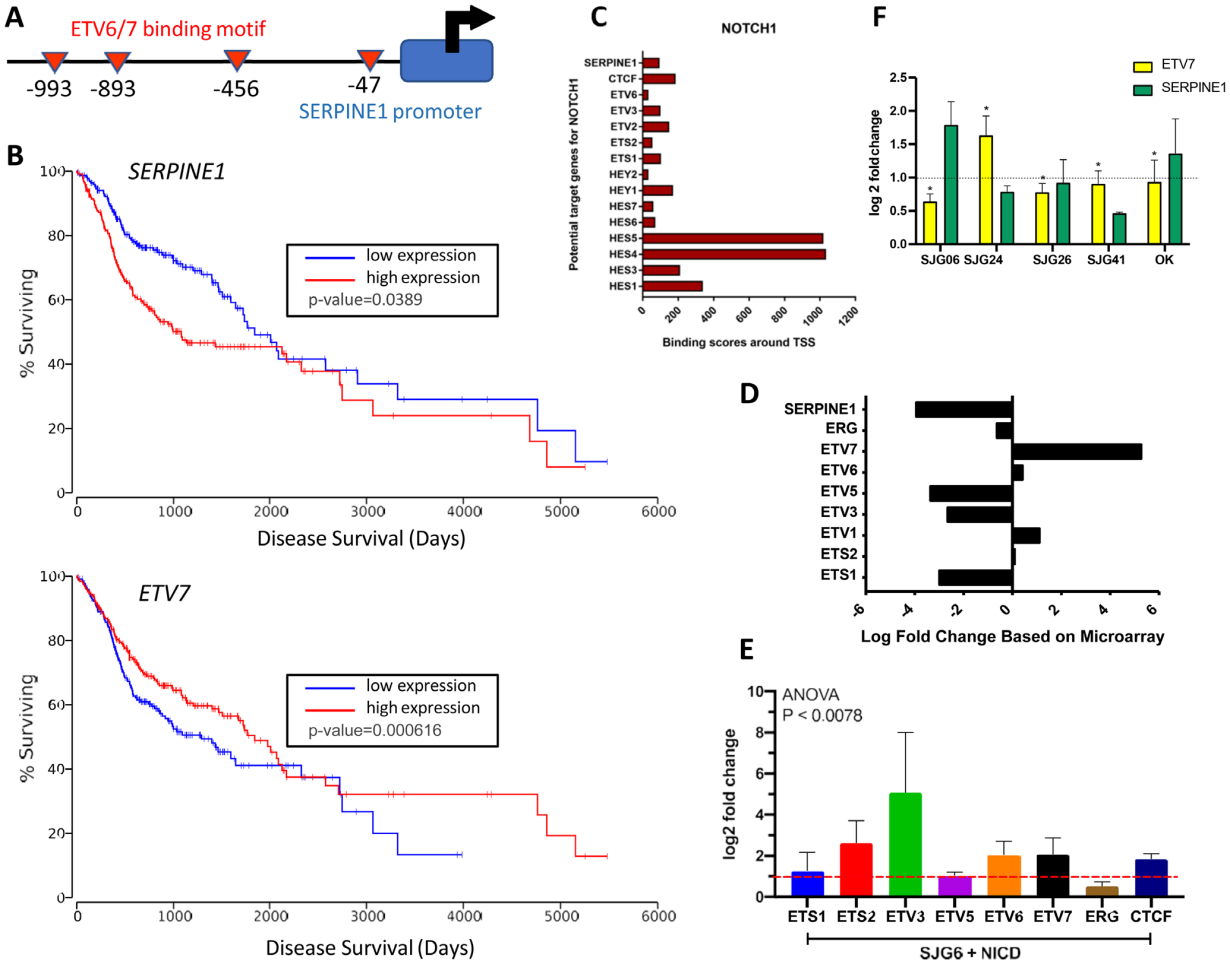
NOTCH1 regulation of SERPINE1 via ETV7. (**A**) Schematic showing ETV6/7 binding site motifs upstream of SERPINE1 promoter. (**B**) Kaplan–Meier survival curves in TCGA-HNSCC patient (*n* = 471) dataset for *SERPINE1* and *ETV7*. Figure reproduced using OncoLnc with cut off of 45.7 [62]. (**C**) ChIP-Atlas data showing efficiency of NOTCH1 binding to ETS and HES/HEY promoters. (**D**) Relative up- and down-regulation of ETS-transcription factors in SJG6+NICD versus SJG6 Blank cells in microarray dataset. (**E**) RT-qPCR in SJG6+NICD versus SJG6 Blank cells (dashed red line) of *CTCF* and *ETS* transcription factors upon NICD overexpression. (**F**) RT-qPCR showing effects of DAPT treatment on *SERPINE1* and *ETV7* expression in oral keratinocytes (OK) and OSCC lines relative to DMSO control (dashed line). *n* = 3. 2-Way ANOVA was used for statistical analysis. (E, F) Data represent mean ± SEM.

To discover whether NICD suppresses *SERPINE1* expression we performed a comparative analysis of the *SERPINE1* promoter region using the Eukaryotic Promoter Database ([32], https://epd.epfl.ch/EPDnew_database.php). We identified multiple *ETV6/7* transcription factor binding motifs upstream of the transcription start site (Figure 5A). The ETS-domain transcription factor family consists of 29 genes in humans and its members are known to either activate or inhibit gene transcription [33]. Most ETS-transcription factors are aberrantly activated or repressed in tumorigenesis [34], and *ETV7*, which encodes TEL2, has been shown to downregulate *SERPINE1* in nasopharyngeal cancer [31]. Examination of the TCGA HNSCC dataset indicated improved survival (*p* = 0.0389) in HNSCC patients with high *ETV7* expression, whereas high *SERPINE1* expression correlated with poor clinical outcome (*p* = 0.000616) (Figure 5B). Thus, *ETV7* and *SERPINE1* emerge as potential predictors of HNSCC prognosis.

According to data deposited in ChIP-Atlas, a comprehensive database for visualizing and exploring public ChIP-seq datasets ([35], https://chip-atlas.org/), NOTCH1 is the only NOTCH protein that binds the transcription start site (TSS) of ETS-transcription factors and *SERPINE1* with comparable binding efficiency to the gene promoter regions of *HES* and *HEY*, two canonical NOTCH1 target genes, (Figure 5C) in T-cell lymphoblastic lymphoma and T-cell Acute Lymphoblastic Leukaemia derived lines [35]. This led us to examine gene expression levels of ETS-transcription factors in the SJG6+NICD versus SJG6 Blank microarray dataset. We observed upregulation of *ETV7* (nearly a 6-fold increase) and downregulation of *ETV5* (almost 4-fold) in SJG6+NICD compared to SJG6 Blank cells (Figure 5D).

To validate these observations experimentally, we performed qRT-PCR. We observed an upregulation of *ETV3* (by 4-fold) and *ETV6/7* (2-fold) in SJG6+NICD compared to SJG6 Blank cells (Figure 5E). To further explore NOTCH1 mediated regulation of *ETV7* and *SERPINE1* expression, NOTCH signaling was inhibited pharmacologically with the γ-secretase inhibitor DAPT. A reduction in *ETV7* and upregulation of *SERPINE1* were observed in both OK and SJG6 (Figure 5F). These results suggest that NICD suppresses *SERPINE1* by upregulation of the ETS-transcription factor ETV7 in OSCC. Nevertheless, the NOTCH pathway is not the sole means by which ETV7 and SERPINE1 are regulated, because in SJG24 cells DAPT treatment led to an increase in ETV7 levels, while in SJG26 and SJG41 cells DAPT did not increase SERPINE1 (Figure 5F).

### Relationship between *NOTCH1, SERPINE1* and *ETV7*/TEL2 expression in OSCC

**Figure 6:**
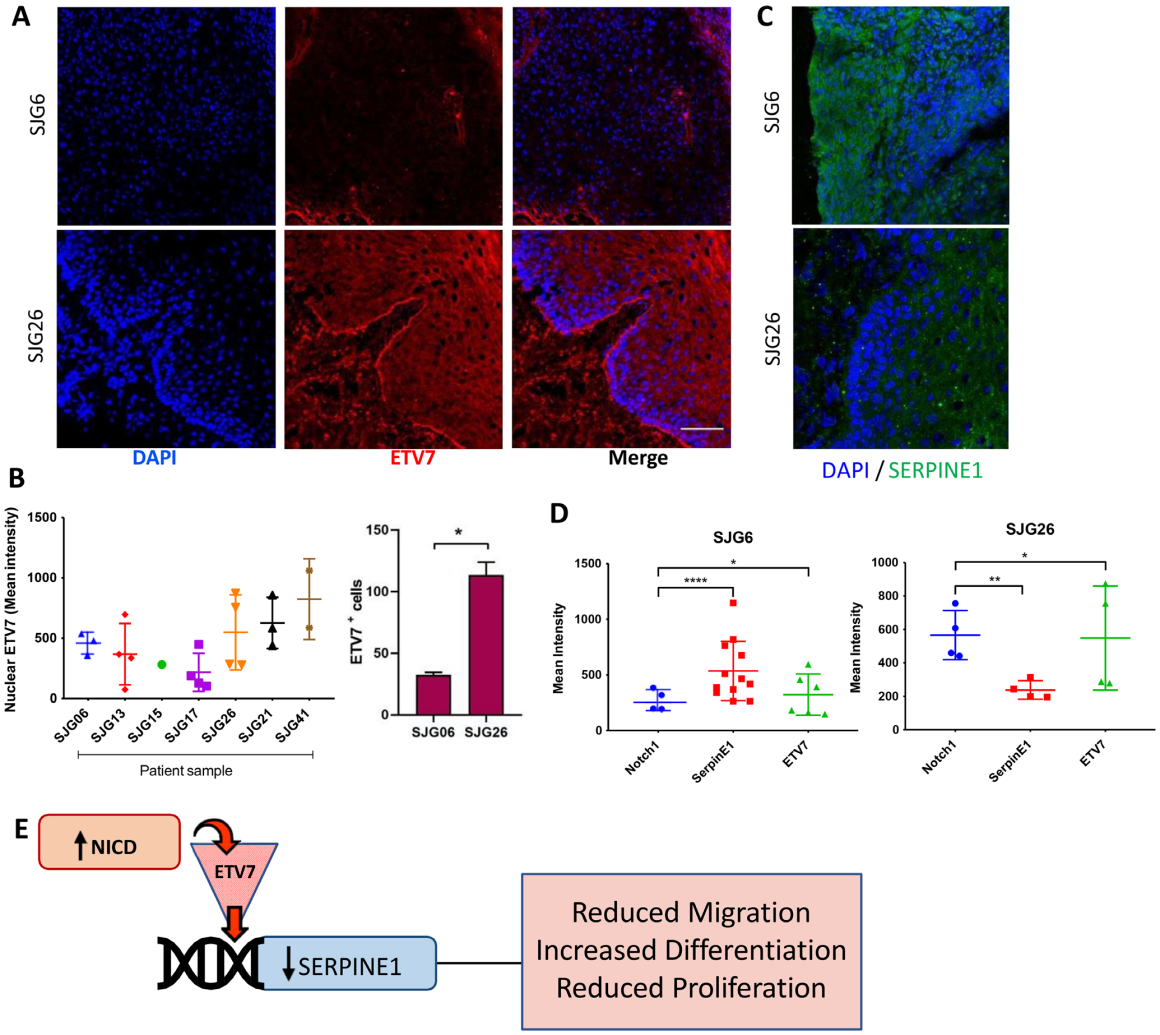
Inverse relationship between *ETV7* and *SERPINE1* in OSCC. (**A**) Representative immunostaining for ETV7 (red) in SJG26 and SJG6 tumour biopsies with DAPI counterstain (blue). Scale bar: 200 μm. (**B**) Quantification of mean nuclear ETV7 staining in OSCC tumour biopsies. Note lower % ETV7 positive cells in NOTCH1 mutant tumour (SJG6) compared to NOTCH1 wild type (SJG26). Multiple *t*-test, ^*^ *p* < 0.05. (**C**) Representative immunostaining for SERPINE1 (green) in SJG26 and SJG6 tumour biopsies with DAPI counterstain (blue). Scale bar: 200 μm. (**D**) Quantification of staining intensities for NOTCH1, ETV7 and SERPINE1 in SJG6 (NOTCH1 mutant) and SJG26 (NOTCH1 WT) tumour biopsies. One sample *t*-test was used. ^*^ *p* < 0.05; ^**^ *p* < 0.01; ^****^ *p* < 0.0001. (**E**) Schematic of the NOTCH1/ETV7/SERPINE1 regulatory axis. (B, D) Data represent mean ± SD.

We next compared the expression levels of *ETV7* and *SERPINE1* in SJG cell lines and the tumours from which they were derived (Figure 6A–6C and Supplementary Figure 1B, 1C). There was very low nuclear ETV7 protein expression in SJG6 (NOTCH1 mutant) compared to SJG26 (NOTCH1 wild type) patient samples, and a significantly lower percentage of ETV7 positive cells (Figure 6B). In addition, *ETV7* expression was inversely correlated with *SERPINE1*, since the SJG6 parental tumour and cell line had higher levels of SERPINE1 than the SJG26 parental tumour and cell line (Figure 6D). Overall, this study suggests a mechanism whereby NICD mediated *SERPINE1* downregulation via ETV7 contributes to several of the tumour-suppressive features of NOTCH1 signaling (Figure 6E).

## Discussion

OSCCs are highly heterogeneous, both genetically and in terms of tumour biology, and have a poor clinical outcome [36]. Hence it is critical to understand the role of specific mutations in determining the properties of individual tumours, as this may lead to the development of new treatments. In the present study we have shown that Notch signaling negatively regulates expression of *SERPINE1* via *ETV7* to control cell behaviour and that *SERPINE1* and *ETV7* correlate inversely with survival in OSCC. This is consistent with an earlier study demonstrating ETV7-mediated downregulation of *SERPINE1* expression and an associated survival benefit in patients with nasopharyngeal carcinoma [31].

The NOTCH1 signaling pathway is highly conserved and regulates diverse cellular processes, including the differentiation of cells in stratified squamous epithelia [37–39]. NOTCH signaling is frequently dysregulated in HNSCCs, often in association with inactivating NOTCH1 mutations [7, 8]. Inhibition of *NOTCH1* in oesophageal epithelial cells leads to clonal expansion, representative of field cancerisation [40]. Although mutational inactivation is frequent in HNSCC [7], other context dependent oncogenic or tumour suppressive roles for NOTCH signaling have been observed in various tumours including HNSCCs [41–43]. This would be consistent with our finding that NOTCH1 mutation is not the sole determinant of NOTCH1 expression in OSCC lines (Figure 1).

To elucidate the mechanism by which NOTCH1 inactivation affects cell behaviour in OSCC, we performed comparative gene expression profiling of SJG6 cells with and without NICD overexpression. NICD rescue led to decreased expression of EMT mediators such as *ZEB1, TWIST1/2, MMP9* and *SNAI2*. This is consistent with evidence suggesting NOTCH1 is a mediator of metastasis via the regulation of *MMP2* and *MMP9* in HNSCC [44]. In addition, NOTCH1 contributes to maintenance of Tumour Necrosis Factor-α (TNF-α)-mediated invasion via transcriptional regulation of *SNAI2* and *TWIST* [45]. Many of the IPA terms in our analysis related *NOTCH1* and *SERPINE1* to cell adhesion, migration, invasion and cell proliferation. A direct interaction between *NOTCH1* and *SERPINE1* in the cell behaviour phenotypes studied in IPA was consistently observed. It has been shown previously that downregulation of *SERPINE1* depends on NOTCH signaling [46]. Most studies have focused on *SERPINE1* upregulation upon wounding, which stimulates epithelial migration [47, 48]. SERPINE1 mediates the response of cell locomotion in *ras*-transformed human keratinocytes treated with TGFβ1 and EGF, and moreover *SERPINE1* upregulation requires the activities of mitogen-activated extracellular kinase (MEK), p21 and pp60 in addition to EGFR [49].

Consistent with the microarray analysis we demonstrated downregulation of *SERPINE1* as a result of NICD overexpression. Overexpression of *SERPINE1* has been related to high risk of metastatic spread in HNSCC [29]. We demonstrated that the changes in cell behaviour and morphology resulting from knockdown of *SERPINE1* resembled those resulting from NICD overexpression, suggesting that *SERPINE1* contributes to the effects of NOTCH1 inactivation. This finding supports a study demonstrating *SERPINE1* downregulation by NICD in differentiated thyroid tumours [30]. *SERPINE1* also protects endothelial cells from FasL-mediated apoptosis [50]. While previous studies have indicated that NICD-induced keratinocyte differentiation is mediated by HES target genes [12, 24], our new results suggest a contribution of ETV7. Although we have not observed cell senescence in our OSCC model, the changes in morphology could be consistent with the senescent phenotype described in OSCC lines [51].

Based on promoter analysis and the ChIP-Atlas database, we identified *ETV7*/*TEL2* as a key factor regulating NICD-mediated *SERPINE1* expression. NICD overexpression in SJG6 cells led to upregulation of several ETS-transcription factors, including *ETV7*, in addition to *HES* transcription factors. Furthermore ChIP-Atlas data confirmed binding of NOTCH1 to the transcription start sites of both ETS-members and *SERPINE1*. Negative regulation of *SERPINE1* by TEL2 in nasopharyngeal carcinoma has already been described [31]. However, regulation of TEL2 by NICD has not been observed previously. ETS-transcription factors regulate a plethora of cellular process, and play a crucial role in tumorigenesis [52]. Recent studies have highlighted the importance of different combinations of ETS- factors in patient stratification and are emerging as novel therapeutic targets in various cancers [53]. Similarly, we observed *ETV7* as a predictor of the patient outcome in HNSCCs.

In summary, our study indicates that the tumour suppressive role of NOTCH1 in OSCC is manifested, at least in part, by *ETV7*-mediated suppression of *SERPINE1*. We have identified the NICD/TEL2/SERPINE1 regulatory axis as a stratification and prognostic tool in OSCC that may be valuable for developing new treatments.

## Materials and Methods

### OSCC biopsies and derivation of OSCC lines

Anonymised biopsies of OSCC or normal oral mucosa were collected with appropriate ethical approval (UK National Research Ethics Service 08/H0306/30), as described previously [13]. The genomic DNA of the 15 SJG cell lines analysed in this study and a further 5 lines for which parental tumour DNA was not available (Supplementary Table 1) was isolated by using the QIAamp DNA Mini Kit (Qiagen). To generate STR profiles of the cells (Supplementary Table 1), PowerPlex assays (Promega) were performed by Source BioScience (Nottingham, UK). The following loci were tested: AMEL, CSF1PO, D13S317, D16S539, D18S51, D21S11, D3S1358, D5S818, D7S820, D8S1179, FGA, Penta D, Penta E, TH01, TPOX, vWA. STR profiles of SJG cells were compared with other cell lines using the STR Similarity Search Tool Cellosaurus 1.1.0 (ExPASy) and found to be distinct.

OSCC lines and a primary oral keratinocyte line (OK) were cultured on a feeder layer of J2 3T3 cells in complete FAD medium as described previously [54]. For some experiments cells were transferred to Keratinocyte Serum Free Medium containing EGF and bovine pituitary extract (KSFM) (Thermo Fisher Scientific).

### Plasmid constructs and transfection

To achieve constitutive expression of NICD, 3xFlagNICD1 (Addgene #20183, [55]) was cloned into the multiple cloning site, under the SFFV promoter, of the LeGO-iC2 plasmid (Addgene#27345, [56]) using the Gateway Vector Conversion System (ThermoFisher Scientific). The plasmids were kind gifts from Raphael Kopan and Boris Fehse, respectively. Lentivirus was produced by transfecting HEK293 with second generation packaging plasmids (psPAX2 - Addgene#12260, and pMD2.G-Addgene#12259) using JetPRIME^®^ (Polyplus Transfection) according to the Manufacturer’s guidelines. OSCC cells were subsequently transduced with lentiviruses.

Transient transfection with SMARTpool ON-TARGETplus SERPINE1 (ID 118572, Thermo Fisher) and Silencer^®^ Negative Control No. 1 (Thermo Fisher) siRNAs was carried out using INTERFERin^®^ (Polyplus transfection).

### Quantitative real-time RT-PCR (qRT-PCR)

RNA isolation and cDNA synthesis were performed using the RNeasy Mini Kit and QuantiTect Reverse Transcription Kit (QIAGEN). qRT-PCR was performed using TaqMan™ Fast Universal PCR Master Mix, no AmpErase™ UNG (Life Technologies) or SYBR Green Master Mix in a Bio-Rad CFX qPCR Instrument with the TaqMan primers listed in Supplementary Table 6. The following qPCR primers were used. NOTCH1: TCCACCAGTTTGAATGGTCA (forward), AGCTCATCATCTGGGACAGG (reverse); NOTCH2: GATCACCCGAATGGCTATGAAT (forward), GGGGTCACAGTTGTCAATGTT (reverse); NOTCH3, TGGCGACCTCACTTACGACT (forward), CACTGGCAGTTATAGGTGTTGAC (reverse); NOTCH4: TGTGAACGTGATGTCAACGAG (forward), ACAGTCTGGGCCTATGAAACC (reverse). Relative quantification of gene expression was performed using 2^-ΔCt^ or 2^-ΔΔCt^ methods.

### Colony formation

72 h after transfection, 500 cells were seeded at single cell density in 6-well plates on a layer of feeders. 10–14 days after seeding, feeders were detached, and keratinocytes were fixed in 10% formalin and stained with a solution of 1% Rhodamine B and 1% Nile blue. Well images were acquired on a Gel Doc™ XR+ (Bio-Rad) and the % of each well covered by colonies was determined using the The ColonyArea plugin tool from ImageJ [57].

### Cell migration

0.5 × 10^6^ cells were seeded per well in KSFM on rat tail collagen I (Corning) - coated 6-well plates. After confluence, cells were treated with 4 μg/ml Mitomycin-C for 2 h. A scratch wound was created and cell migration into the wound area was monitored for 20 h using an IncuCyte^®^ZOOM (Essen BioScience). Relative wound area was measured using ImageJ.

### Cell proliferation, differentiation and morphology

To assay proliferation, cells were seeded in KSFM at a concentration of 10^5^ cells/well in collagen-coated 96-well plates. Cell proliferation was determined with a Click-iT Alexa Fluor 488 Imaging Kit (Life Technologies).

For morphology and differentiation assays, cells were seeded at clonal density in 12-well plates coated with collagen for the differentiation assay or fibronectin (0, 1, 5, 25 μg/ml) to measure cell morphology. After 72 h cells were fixed in 4% paraformaldehyde, permeabilised in 0.1% Triton™ X-100 in PBS for 5 min, and stained with DAPI (Thermofisher, D1306) and Phalloidin (detailed in Supplementary Table 7) for 1 h at room temperature or labelled overnight at 4°C with anti-involucrin (SY7 [58]) antibody, followed by 1 h incubation with Alexa Fluor-conjugated Secondary Antibody at room temperature (RT). Details of all the Primary and secondary antibodies used in the study are listed in Supplementary Table 7. Images were acquired using the Operetta^®^ High-Content Imaging System (PerkinElmer). Cell morphology features (cell area, elongation and roundness), % EdU and % Involucrin positive cells were calculated using Harmony Software (PerkinElmer).

### Pharmacological inhibition of Notch signaling

The γ-secretase inhibitor N-[N-(3,5-Difluorophenacetyl)-L-alanyl]-S-phenylglycine t-butyl ester (DAPT; Sigma), dissolved in dimethyl sulfoxide (DMSO; Stratech), was used to block Notch signaling. Cells were seeded in 6-well plates at 10^5^ cells per well in 2 ml KSFM supplemented with 10 µM DAPT or DMSO. After 24 h cells were grown in complete FAD medium for 48 h and then harvested.

### Gene expression microarrays

cDNA was fragmented and labelled with the Affymetrix GeneChip Labeling Kit, followed by hybridization on Human Gene 1.0 ST Arrays (Affymetrix). The microarrays were scanned on a GeneChip scanner 3000 (Affymetrix) and analysed using the oligo (Carvalho and Irizarry, 2010) and limma [59] packages in R-studio software (https://rstudio.com/). Raw data, in CEL format, were normalized using Robust Multichip Average (RMA) and annotated using the Entrez IDs from the ‘hugene20transcripcluster’ package. A heatmap using the ‘gplots’ package [60] was then created for the differentially expressed genes filtered at a *p*-value of 0.05 and log-fold change of 2. Go-Term and GSEA were run prior to exporting the gene list and fold change values to Ingenuity Pathway Analysis (IPA) Software. The data are deposited in Gene Expression Omnibus (accession number GSE127770).

### Immunofluorescence microscopy

Frozen 6–8 μm sections were fixed in 4% paraformaldehyde and permeabilised in 0.5% Triton™ X-100 in PBS at room temperature or with 1:1 acetone-methanol (for nuclear antigens) at –20°C for 5 min. Sections were blocked with a solution of 10% donkey serum, 0.1% fish skin gelatin, 0.1% Triton X-100, and 0.5% Tween-20 (all from Sigma) in phosphate buffered saline (PBS), and labelled overnight at 4°C with primary antibodies (Supplementary Table 7) diluted in blocking buffer. Sections were washed with PBS and then labelled with Alexa Fluor-conjugated secondary antibodies and DAPI for 1 h at RT, washed with PBS, and mounted with ProLong Gold antifade reagent (Thermo Fisher Scientific). Confocal microscopy was performed with a Nikon A1 Upright Confocal microscope (Tokyo, Japan) using 20× objectives. Image processing was performed with Image J (Fiji) (National Institutes of Health, Bethesda, MD).

### Western blotting

30 μg protein were loaded into pre-cast 4–15% Mini-PROTEAN^®^ TGXTM gels (Bio-Rad) alongside Precision Plus Protein™ Dual Colour Standards (Bio-Rad) and run at 150V in Tris/Glycine/SDS Running Buffer (Bio-Rad). The Trans-Blot^®^ Turbo™ Transfer Starter System, Mini PVDF (Bio-Rad) was used to transfer protein to membranes followed by blocking for 1 h, overnight incubation with primary antibodies at 4°C and 1 h incubation with horseradish peroxidase (HRP)-conjugated secondary antibody at RT. ClarityTM ECL Western Blotting Substrate (Bio-Rad) and ChemiDoc Touch (Bio-Rad) were used to image the membranes. Relative protein band intensities were quantified and normalized against Vinculin using densitometric analysis.

### Statistical analysis

Graphs were generated using GraphPad Prism7 (La Jolla, CA) and represent Mean ± SEM of 3 independent experiments. One-way analysis of variance parametric test with Bonferroni post-test or Student’s *t*-test was performed, with *p* < 0.05 considered significant.

## SUPPLEMENTARY MATERIALS









## Abbreviations

DAPT: (2S)-N-[(3,5-Difluorophenyl)acetyl]-L-alanyl-2-phenyl]glycine1,1-dimethylethylester
DMSO: Dimethyl Sulfoxide
ECM: Extracellular Matrix
GSEA: Gene Set Enrichment Analysis
HNSCC: Head and Neck Squamous Cell Carcinoma
KEGG: Kyoto Encyclopaedia of Genes and Genomes
NICD: Notch Intracellular Domain
NECD: Notch Extracellular Domain
OSCC: Oral Squamous Cell Carcinoma
OK: Oral Keratinocytes
