# Supplementary material for: NOTCH1 signaling in oral squamous cell carcinoma via a TEL2/SERPINE1 axis
Source: Oncotarget. 2019 Nov 26;10(63):6791–804. doi: 10.18632/oncotarget.27306 (PMC6887571; doi:10.18632/oncotarget.27306)
Supplement: Supplementary file 1 [file oncotarget-10-6791-s001.pdf]

NOTCH1 signaling in oral squamous cell carcinoma via a TEL2/ SERPINE1 axis

SUPPLEMENTARY MATERIALS

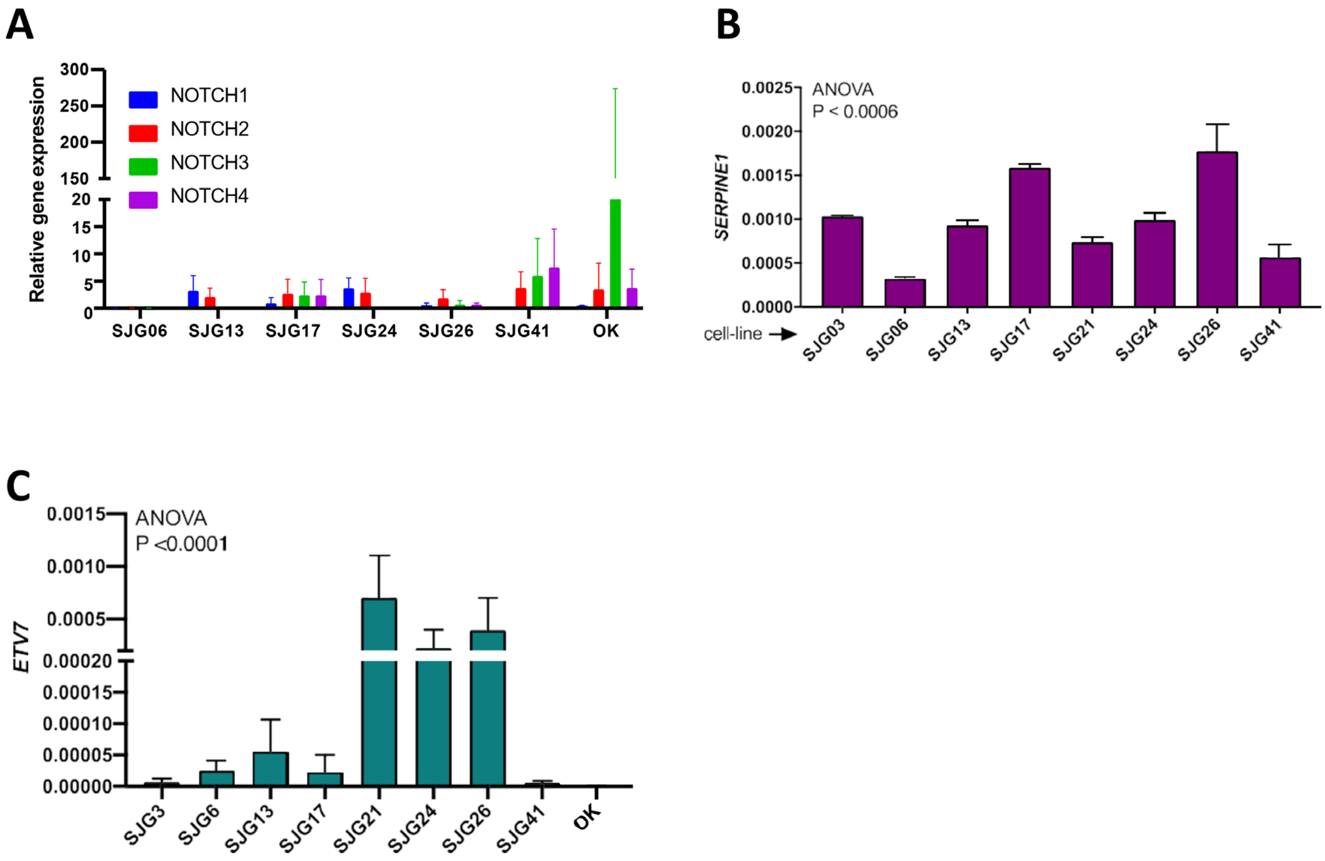

Supplementary Figure 1: Expression of NOTCH, SERPINE1 and ETV7 in OSCC lines. (**A**) RT-qPCR showing relative expression of NOTCH *1, 2, 3* and *4* in SJG lines and OK, *n* = 3. (**B**) RT-qPCR analysis showing levels of *SERPINE1* mRNA in SJG lines, *n* = 3. (**C**) RT-qPCR analysis of levels of ETS1, ETS2, ETV6 and ETV7 mRNA in SJG lines and OK, *n* = 3. Data represent mean ± SD.

Supplementary Table 1: STR profiles of SJG lines and validation of NOTCH1 mutations by Sanger sequencing. See Supplementary Table 1

Supplementary Table 2: Microarray data for SJG6 Blank and SJG+NICD cells. See Supplementary Table 2

Supplementary Table 3: KEGG pathway analysis

Pathway Name	Corrected *p* value	log value
ECM-receptor interaction	4.61E-05	4.336299075
Focal adhesion	0.0002	3.698970004
Notch signaling pathway	0.0008	3.096910013
Regulation of actin cytoskeleton	0.0011	2.958607315
Metabolic pathways	0.0037	2.431798276
p53 signaling pathway	0.0037	2.431798276
Insulin signaling pathway	0.0037	2.431798276
Tight junction	0.0098	2.008773924
Hypertrophic cardiomyopathy (HCM)	0.0102	1.991399828
Tyrosine metabolism	0.0105	1.978810701
Fatty acid metabolism	0.0119	1.924453039
Leukocyte trans endothelial migration	0.0131	1.882728704
Viral myocarditis	0.0161	1.793174124
Cell cycle	0.0168	1.774690718
Toll-like receptor signaling pathway	0.0197	1.705533774
Axon guidance	0.0197	1.705533774
Amoebiasis	0.0228	1.642065153
Other glycan degradation	0.0228	1.642065153
Protein digestion and absorption	0.0228	1.642065153
Progesterone-mediated oocyte maturation	0.0292	1.534617149
Apoptosis	0.0292	1.534617149
Vascular smooth muscle contraction	0.0292	1.534617149
alpha-Linolenic acid metabolism	0.0296	1.528708289
Dilated cardiomyopathy	0.0306	1.514278574
Fat digestion and absorption	0.0358	1.446116973
Other types of O-glycan biosynthesis	0.0358	1.446116973
Spliceosome	0.0358	1.446116973
Fatty acid elongation in mitochondria	0.0358	1.446116973
Fc gamma R-mediated phagocytosis	0.0358	1.446116973
Bacterial invasion of epithelial cells		
Purine metabolism	0.0358	1.446116973
MAPK signaling pathway	0.0358	1.446116973
Adipocytokine signaling pathway	0.0362	1.441291429

Arrhythmogenic right ventricular cardiomyopathy (ARVC)	0.0362	1.441291429
Toxoplasmosis	0.0362	1.441291429
Type II diabetes mellitus	0.0362	1.441291429
Cell adhesion molecules (CAMs)	0.0437	1.359518563
Phosphatidylinositol signaling system	0.0495	1.305394801
Pathways in cancer	0.0502	1.299296283
Butanoate metabolism	0.0502	1.299296283
Citrate cycle (TCA cycle)	0.053	1.27572413
Pathogenic Escherichia coli infection	0.053	1.27572413
Huntington's disease	0.055	1.259637311
Arachidonic acid metabolism	0.0604	1.218963061
Lysosome	0.0605	1.218244625
Ether lipid metabolism	0.0715	1.145693958
Osteoclast differentiation	0.0741	1.130181792
GnRH signaling pathway	0.0882	1.054531415
Pyruvate metabolism	0.0882	1.054531415
Ubiquitin mediated proteolysis	0.0882	1.054531415
PPAR signaling pathway	0.089	1.050609993
Long-term depression	0.089	1.050609993
RIG-I-like receptor signaling pathway	0.0912	1.040005162
Leishmaniosis	0.0936	1.028724151
Chronic myeloid leukaemia	0.0961	1.017276612
Lysine degradation	0.0987	1.005682847

Supplementary Table 4: GO terms

Go Accession	GO Term	*p*-value	Corrected *p*-value	Count in Selection	% Count in Selection	Count in Total	% Count in Total
GO:0031428	box C/D snoRNP complex	4.14E-10	9.78E-06	5	4.9504952	9	0.046066437
GO:0070761	pre-snoRNP complex	2.57E-09	3.04E-05	5	4.9504952	12	0.061421916
GO:0005732	small nucleolar ribonucleoprotein complex	4.87E-08	3.83E-04	5	4.9504952	20	0.10236986
GO:0030515	snoRNA binding	8.21E-08	4.84E-04	5	4.9504952	22	0.112606846
GO:1902904	negative regulation of fibril organization	1.34E-07	5.28E-04	3	2.970297	3	0.015355479
GO:1902903	regulation of fibril organization	1.34E-07	5.28E-04	3	2.970297	3	0.015355479
GO:0030308	negative regulation of cell growth	1.89E-06	0.006361553	8	7.920792	161	0.82407737
GO:0005615	extracellular space	2.58E-06	0.00695763	21	20.79208	1303	6.6693964
GO:0000974	Prp19 complex	2.65E-06	0.00695763	3	2.970297	6	0.030710958
GO:1903054	negative regulation of extracellular matrix organization	4.62E-06	0.010325498	3	2.970297	7	0.03582945
GO:0044421	extracellular region part	4.81E-06	0.010325498	39	38.61386	3765	19.271126
GO:0042026	protein refolding	6.60E-06	0.012991914	4	3.960396	24	0.12284383
GO:0005576	extracellular region	7.22E-06	0.013113523	44	43.564358	4606	23.575779
GO:0051346	negative regulation of hydrolase activity	8.69E-06	0.014658065	11	10.891089	409	2.0934637
GO:0044452	nucleolar part	1.13E-05	0.016684284	5	4.9504952	57	0.2917541
GO:0008201	heparin binding	1.13E-05	0.016684284	7	6.930693	147	0.75241846
GO:0005539	glycosaminoglycan binding	1.29E-05	0.01796228	8	7.920792	209	1.0697651
GO:0071675	regulation of mononuclear cell migration	2.15E-05	0.028164886	3	2.970297	11	0.056303423
GO:0030336	negative regulation of cell migration	2.89E-05	0.032474402	7	6.930693	170	0.87014383
GO:0031932	TORC2 complex	2.85E-05	0.032474402	3	2.970297	12	0.061421916
GO:2000506	negative regulation of energy homeostasis	2.65E-05	0.032474402	2	1.980198	2	0.010236986
GO:0045926	negative regulation of growth	3.10E-05	0.032977507	8	7.920792	236	1.2079644
GO:0002688	regulation of leukocyte chemotaxis	3.32E-05	0.032977507	5	4.9504952	71	0.363413
GO:2000146	negative regulation of cell motility	3.35E-05	0.032977507	7	6.930693	174	0.8906178
GO:0051085	chaperone mediated protein folding requiring cofactor	3.69E-05	0.034886237	3	2.970297	13	0.06654041
GO:0061202	clathrin-sculpted gamma-aminobutyric acid transport vesicle membrane	4.68E-05	0.04095736	3	2.970297	14	0.0716589

GO:0061200	clathrin-sculpted gamma-aminobutyric acid transport vesicle	4.68E-05	0.04095736	3	2.970297	14	0.0716589
GO:0038201	TOR complex	5.83E-05	0.04918293	3	2.970297	15	0.0767774
GO:0051271	negative regulation of cellular component movement	6.06E-05	0.04933887	7	6.930693	191	0.97763216
GO:0040013	negative regulation of locomotion	6.69E-05	0.052617405	7	6.930693	194	0.9929877
GO:0070704	sterol desaturase activity	7.91E-05	0.056610905	2	1.980198	3	0.015355479
GO:0000248	C-5 sterol desaturase activity	7.91E-05	0.056610905	2	1.980198	3	0.015355479
GO:2001170	negative regulation of ATP biosynthetic process	7.91E-05	0.056610905	2	1.980198	3	0.015355479
GO:1903053	regulation of extracellular matrix organization	8.65E-05	0.05994675	3	2.970297	17	0.087014385
GO:1901681	sulfur compound binding	8.89E-05	0.05994675	7	6.930693	203	1.0390542
GO:0001558	regulation of cell growth	1.02E-04	0.064264275	9	8.910892	359	1.8375391
GO:0060198	clathrin-sculpted vesicle	1.03E-04	0.064264275	3	2.970297	18	0.092132874
GO:0002040	sprouting angiogenesis	1.01E-04	0.064264275	4	3.960396	47	0.24056917
GO:0050921	positive regulation of chemotaxis	1.21E-04	0.07151497	5	4.9504952	93	0.47601986
GO:0051607	defense response to virus	1.20E-04	0.07151497	6	5.940594	148	0.757537
GO:0023023	MHC protein complex binding	1.43E-04	0.077097766	3	2.970297	20	0.10236986
GO:0030334	regulation of cell migration	1.41E-04	0.077097766	11	10.891089	556	2.8458822
GO:0023026	MHC class II protein complex binding	1.43E-04	0.077097766	3	2.970297	20	0.10236986
GO:0031324	negative regulation of cellular metabolic process	1.44E-04	0.077097766	24	23.762377	2104	10.769309
GO:2001169	regulation of ATP biosynthetic process	1.58E-04	0.08274892	2	1.980198	4	0.020473972

Supplementary Table 5: GSEA molecular functions. See Supplementary Table 5

Supplementary Table 6: List of TaqMan® probes

TaqMan probes	Company/Source	Assay ID
NOTCH1	Thermofisher	Hs01062014_m1
SERPINE1	Thermofisher	Hs00167155_m1
ETS1	Thermofisher	Hs00428293_m1
ETS2	Thermofisher	Hs00232009_m1
ETV3	Thermofisher	Hs01051028_g1
ETV5	Thermofisher	Hs00927557_m1
ETV6	Thermofisher	Hs00231101_m1
ETV7	Thermofisher	Hs00903229_m1
ERG	Thermofisher	Hs01554629_m1
CTCF	Thermofisher	Hs00902016_m1
18S	Thermofisher	Hs03003631_g1
TBP	Thermofisher	Hs00427620_m1

Supplementary Table 7: List of primary and secondary antibodies used for immunofluorescence and Western blot

Antibody	Company/Source	Catalog number/Reference	Host species	Dilution IF or (WB)
Phalloidin AF488	Thermofisher	A12379	NA	1/2000
Phalloidin AF555	Thermofisher	A34055	NA	1/2000
Phalloidin AF647	Thermofisher	A22297	NA	1/2000
anti-Cleaved NOTCH1 (Val1744)	Cell Signaling Technology	D3B8 #4147	Rabbit	1/100
anti-SERPINE1 (1D5)	Novus Biologicals	NBP2-37532	Mouse	1/100, 1/250 (WB)
anti-ETV7	Atlas Antibodies	HPA029033	Rabbit	1/100
anti-Involucrin	In-house	Sy7 Hudson D, et al., Hybridoma (1992), 11(3):367-379	Mouse	1/500
anti-Mouse AF555	Life Technologies	A31570	Mouse	1/300
anti-Mouse AF647	Life Technologies	A31571	Mouse	1/300
anti-Mouse AF488	Life Technologies	A11001	Mouse	1/300
anti-Rabbit AF488	Life Technologies	A11008	Rabbit	1/300
anti-Rabbit AF555	Life Technologies	A21428	Rabbit	1/300
anti-Rabbit AF647	Life Technologies	A21244	Rabbit	1/300
anti-Vinculin (7F9)	Santa Cruz Biotechnology	sc-73614	Mouse	1/10000 (WB)
anti-Mouse HRP	Jackson Lab	715-035-151	Mouse	1/10000 (WB)

Footnote: NA-Not applicable; IF- Immunostainings of cells or tissue; WB- Western blotting.
